# Fabrication of Nanostructures on a Large-Area Substrate with a Minimized Stitch Error Using the Step-and-Repeat Nanoimprint Process

**DOI:** 10.3390/ma15176036

**Published:** 2022-09-01

**Authors:** Yeonjoo Ha, Hyungjun Lim, Hak-jong Choi, JaeJong Lee

**Affiliations:** 1 Korea Institute of Machinery and Materials, 156, Gajeongbuk-ro, Yuseong-gu, Daejeon 34103, Korea; 2 Department of Nanomechatronics, University of Science and Technology, 217, Gajeong-ro, Yuseong-gu, Daejeon 34113, Korea

**Keywords:** nanoimprint lithography, large-area nanostructures, stitch error, step-and-repeat nanoimprint process, nanopatterns, stamp

## Abstract

Nanoimprint lithography (NIL) is suitable for achieving high uniformity and mass production. However, in conventional NIL, a stamp suitable for the substrate size is required to increase the substrate size. To address this issue, we fabricated nanostructures on a large-area substrate using step-and-repeat NIL after making a small stamp. A stamp was produced using glass, and a nano-pillar pattern with a diameter of 600 nm, an interval of 400 nm, and a height of 270 nm was used during the experiment. The area of the pattern on the stamp was 10 mm × 10 mm, and the step-and-repeat process was performed 25 times to transfer the nanostructures to a 4-inch substrate. In addition, stitch gaps were created between the patterns, which could decrease the performance upon future application. To minimize this stitch gap, a high-precision glass scale was attached to the stamp feeder to precisely control the position and to minimize the step difference. Moreover, an experiment was conducted to minimize the stitch gap by adjusting the movement interval of the stamp, and the stitch spacing was minimized by moving the stamp position by 9.97 mm. This approach will facilitate the manufacturing of large-area substrates and other structures in the future.

## 1. Introduction

Nanostructures are fabricated using various techniques, such as electron beam li-thography [1], photolithography [2], nanoimprint lithography (NIL) [3], and soft lithog-raphy [4]. Among these techniques, NIL can stably fabricate nanostructures with high uniformity at low cost. NIL transfers the nanostructure to a substrate using heat or ultra-violet (UV) light while applying a constant pressure, according to the polymer curing method [5]. NIL processes are used in various applications, such as displays, solar cells, and flexible electronic devices [6]. Recently, developing a method of transferring nanostructures to large-area substrates has emerged as an important issue [7]. In the past, for large-area structures, it was necessary to produce a stamp that suited the size of the substrate. However, producing a stamp with a functional pattern on a large-area substrate presents challenges in terms of cost and manufacturing. To address these shortcomings, the roll-to-roll and step-and-repeat processes can be used to transfer nanostructures to large-area substrates. The roll-to-roll process is a method of manufacturing a stamp with polydimethylsiloxane, in which the substrate is attached to each roll using a film, for example, and then the nanostructure is transferred to a large-area substrate [8]. In the roll-to-roll process, even if the size of the stamp is not large, it is possible to transfer the nanostructure to a large-area substrate by repeatedly performing the process. However, as the substrate must be attached to the roll, the substrate must be composed of a flexible material. Other methods of transferring a nanostructure to a large area are the step-and-repeat method and the step-and-flash imprint method [9,10]. In order to use a stamp in the step-and-repeat process, it is very important to sharpen the edge of the stamp to minimize hardening of the boundary during stamp production.

The existing step-and-repeat NIL method may cause problems, such as damage to the stamp due to the repetition of the process, and the pattern must be well-transferred during the process. In addition, in recent years, minimizing the stitching gap has emerged as an important concern. A stitch refers to an area of the pattern that cannot be transferred in the vertical and horizontal directions [11]. Furthermore, there are several types of stitch errors that indicate errors due to stitch gaps [12]. The stitch is caused by hardening resistance in the non-patterned area due to an error in the transfer system during stamp manufacturing and the leakage of UV light. Stitch spacing also affects the subsequent steps of the step-and-repeat process. If a perfect pattern is not formed in the subsequent process, there is a high probability that the device performance will decrease when it is manufactured later [13]. Therefore, it is imperative to minimize the stitch gap while transferring the nanostructures onto a large-area substrate. To minimize the stitch gap, it is necessary to produce a stamp with clear edges to ensure complete contact between the substrate and stamp and to irradiate the UV light in parallel to harden the resin on the contacted side.

In this paper, we propose a process for transferring nanostructures to a substrate using a small-sized stamp and the step-and-repeat NIL process. In addition, an experiment was conducted to minimize the stitch gap that may occur during the process. Firstly, small-sized stamps were produced using glass. Glass was used to allow UV light to pass through and to cure the UV resin; the pattern area was 10 mm × 10 mm, and a nanopillar structure with a width of 600 nm was fabricated over the entire area. During the step-and-repeat process, in order to apply pressure only when the region of the pattern comes into contact with the substrate, a step difference of 5 μm was created during stamp manufacturing. In addition, chromium was deposited to prevent UV light from leaking into areas without a pattern during pattern transfer. In order to conduct an experiment to reduce the stitch gap, the edge of the pattern area was made sharper when making the stamps. In addition, the UV light in the system was designed to fall vertically and uniformly. The system used in this experiment was the ANT-520SUV step-and-repeat NIL system developed in our laboratory to transfer nanostructures onto a 4-inch substrate using a small stamp.

## 2. Materials and Methods

### 2.1. Stamp Fabrication

**Figure 1:**
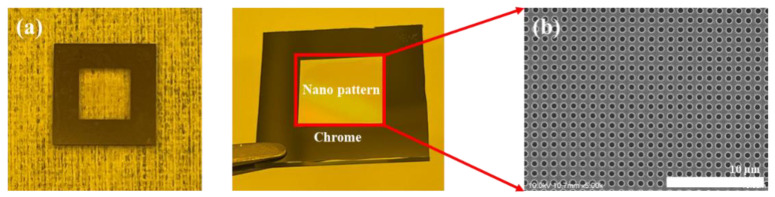
(**a**) Nanopattern stamp with deposited chromium and (**b**) 600 nm diameter nanohole structure.

Firstly, a glass master with a pillar pattern, a 600 nm diameter, 400 nm intervals, and a 270 nm height was fabricated using a stepper. The reason that glass was used as a master was to enable UV light to pass through and contact the UV resin. During the process, a stamp was produced so that the pattern area was raised by 5 μm, in order to ensure that only the pattern area was uniformly in contact with the substrate. In addition, chromium was deposited to a thickness of 20 nm in areas other than the pattern through chemical vapor deposition to prevent the resin from curing in areas other than the pattern when UV light is emitted. Figure 1 shows the fabricated stamp and finished nanostructure pattern. The fabricated glass stamp was coated with 1H,1H,2H,2H-Perfluorooctyltriethoxysilane (Sigma-Aldrich, St. Louis, MO, USA) as an anti-adhesion coating. The fabricated small stamp was attached to the glass backbone using UV adhesive, and the fabricated stamp attached to the glass backbone was connected to a chuck in the system and utilized in the step-and-repeat NIL process.

### 2.2. Step-and-Repeat Process

**Figure 2:**
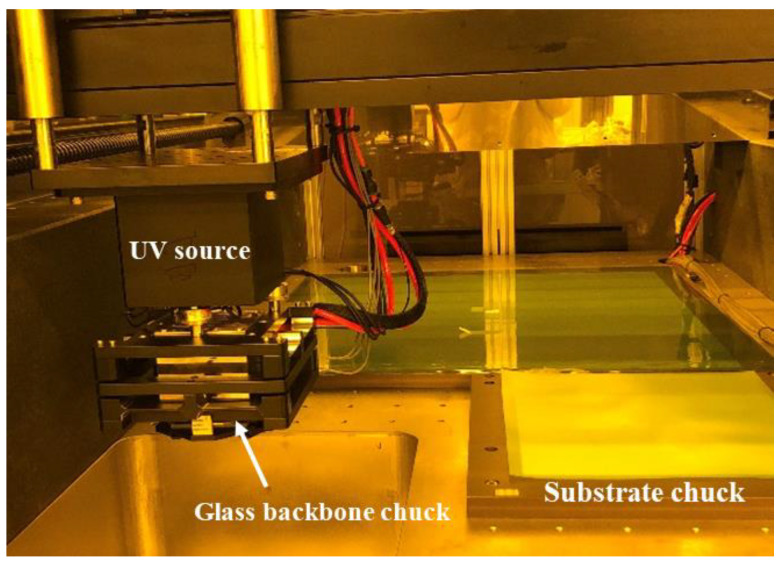
Step-and-repeat NIL system (ANT-520SUV developed by KIMM).

The step-and-repeat NIL system (ANT-520SUV, developed by KIMM, Jang-dong, Korea) employed in this study supports a substrate size of 500 mm × 200 mm and was designed to enable the loading of wafers and piece specimens. The stamp with the manufactured nanopattern was attached to the glass backbone and then chucked into the system. The stamp chuck unit uses a flexible mechanism for uniform contact between the stamp pattern area and substrate, even at low pressures of 98 N/cm^2^ or less, and the stamp and substrate were subjected to uniform pressure. The ANT-520SUV device employs a UV unit with a wavelength of 365 nm. The XY-axis stage utilizes a glass scale with a resolution higher than 1 µm and a linear motion guide to enable positioning control, with a positioning accuracy of 2 µm and repeatability accuracy of 1 µm. The Z-axis stage was designed to control the position at the 2 µm level using encoder pulses. Figure 2 shows the equipment used in this experiment.

In order to proceed with the process, the entire area of the silicon substrate was coated with UV resin. After attaching the glass stamp with the nanopattern to the chuck, the pressure for the process was input. When the set pressure was reached after the silicon substrate and the stamp were in contact, UV curing occurred on the contact surface. After the first process was completed, the substrate and stamp were demolded, the stamp was moved at regular intervals, and the curing and demolding process was repeated under the same conditions as in the previous process. In this experiment, one stamp was used, and the process was performed a total of 25 times.

### 2.3. Experimental Conditions

**Figure 3:**
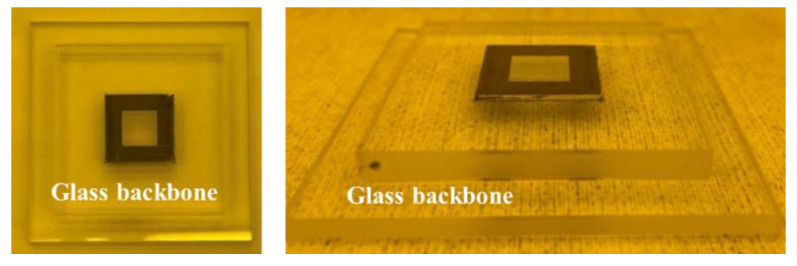
A stamp is attached to the glass backbone, which is required for attachment of equipment.

Figure 3 shows the stamp employed in this experiment attached to the glass back-bone using a UV adhesive. The finished stamp was attached to the equipment to facilitate movement. A 4-inch silicon wafer and a polyethylene terephthalate (PET) film with a thickness of 0.2 mm and 140 mm × 60 mm in size were utilized as the substrate. Polyurethane acrylate (PUA, Changsung Sheet), a UV resin, was spin-coated onto the silicon substrate at a speed of 3000 rpm for 30 s. After loading the coated substrate onto the substrate chuck in the equipment, the step-and-repeat process was performed. The equipment was designed to apply constant pressure to the stamp and substrate even if the pressure is low. Accordingly, an experiment was conducted to set a pressure of 19 N/cm^2^ during the process. When the stamp applies a pressure of 19 N/cm^2^ to the substrate, the UV light turns on and cures for 60 s. Due to the chromium deposition of the stamp, curing of the UV resin occurred only in the pattern area, allowing the step-and-repeat process to proceed repeatedly. After the curing process had been completed, the stamp was demolded from the substrate and the stamp stage was moved. The stage was moved by 10 mm, 9.97 mm, or 9.95 mm, and the process was repeated under the same conditions using one stamp, according to the set stage movement value.

**Figure 4:**
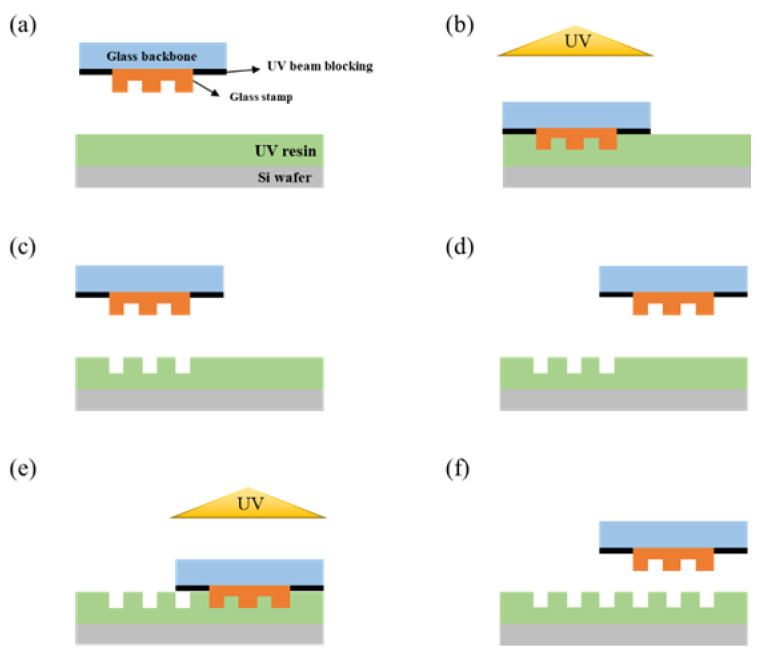
Schematic of the step-and-repeat NIL process. (**a**) The substrate is coated with resin, and the stamp is attached to the chuck. (**b**) When the pattern area and substrate are in contact, UV is irradiated, and the pattern is transferred to the substrate. (**c**) After UV irradiation, the demolding process takes place. (**d**) After the stamp is demolded, the stamp moves by the set value. (**e**) After the stamp moves by the set value, UV is irradiated when it contacts the substrate again. (**f**) After UV irradiation, the stamp is demolded, and the process is repeated.

Figure 4 shows a schematic diagram of the process conducted in the experiment. After coating the entire substrate with PUA, a UV resin, when the first process is finished, the stamp moves by as much as the set value to proceed with the next process. In Figure 4, chromium is deposited to block the UV beam so that the area other than the pattern is not cured.

## 3. Results

**Figure 5:**
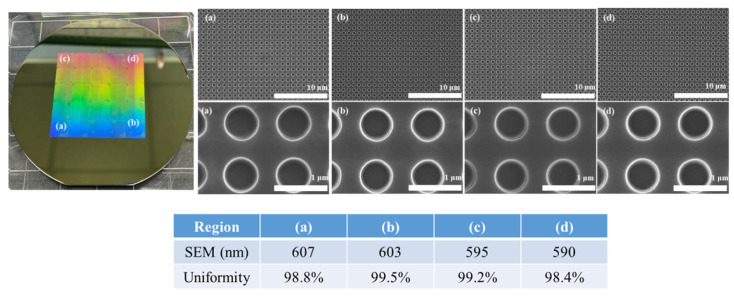
Results of the step-and-repeat NIL on the 4-inch silicon wafer and the diameter uniformity of the hole pattern in the same four regions after the step-and-repeat NIL.

**Figure 6:**
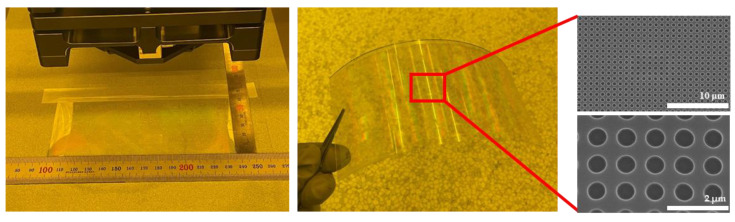
Results of the step-and-repeat NIL on the PET film.

Figure 5 shows the results of transferring the pattern to a 4-inch silicon substrate by repeating the position movement and curing a total of 25 times using a 10 mm × 10 mm stamp. Comparing the nanostructures of the master stamp shown in Figure 1 and the nanostructures transferred to the substrate after processing, it was confirmed that there was no difference in the shape of the pattern. In addition, the shape of the pattern transferred to the substrate was compared by repeating the process 25 times under the same conditions. When the pattern shapes were compared by selecting four regions of the substrate after the process had been completed, there was no difference in the shape of the patterns, as shown in Figure 5, and it was confirmed that the nanostructures were uniformly transferred. Consequently, it was confirmed that the large-area patterning process was possible because the pattern shape of the stamp was not affected, even if the process was performed several times using one small stamp. In addition, Figure 6 shows the result of transferring the pattern to the PET film substrate through a repeat process. As shown in Figure 6, it was found that pattern transfer was possible after the process was carried out, even when the film was used as a substrate, with a size of 140 mm × 60 mm.

When the step-and-repeat imprint process is performed, a stitch gap is created between the patterns because the edge part of the pattern is cured in advance. In this study, three methods were used to reduce the stitch gap. The first method involved reducing the step difference between the patterned and non-patterned areas when fabricating the stamps. Reducing the step difference during stamp production reduces the amount of light leaking through the side, minimizing the possibility of pre-curing in areas other than the patterned area. If the possibility of pre-curing is minimized, then the subsequent process will be less affected; therefore, the process can be performed while minimizing the stitch gap. The stamp produced for this experiment was processed with a step difference of approximately 5 μm to minimize UV light leakage via the side of the stamp.

In the second method, beam blocking was performed on the non-patterned area of the stamp using the chromium deposition process. By blocking all areas other than the patterned area of the stamp, the UV light was blocked, and curing occurred only in the area in which the pattern and substrate were in contact, minimizing the effect of the pre-cured resin during the step-and-repeat nanoimprint process.

Finally, a glass scale was attached to the linear motion guide of the XY-stage of the ANT-520SUV device to reduce the stitch gap by lowering the position error due to the XY-axis transfer by up to 1 µm. By attaching a high-precision glass scale to the feed system of the XY-stage, the stitch gap could be reduced when the stamp was moved, before proceeding to the second process. In addition, the equipment was designed so that UV light would fall vertically and only the pattern area would be cured by UV light. The vertically dropped UV light did not pre-cure the non-patterned area, so the stitch gap could be minimized.

**Figure 7:**
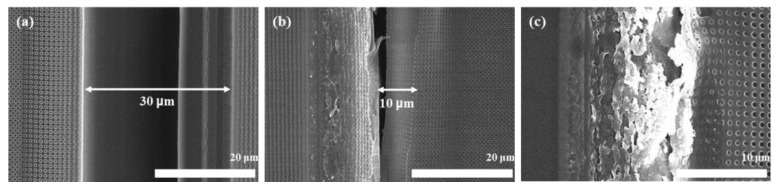
Stitch gap displacement by (**a**) 10 mm, (**b**) 9.97 mm, and (**c**) 9.95 mm.

Figure 7a–c show the results when the stamp stage is displaced by 10, 9.97, and 9.95 mm, respectively, during the process. When the stage is initially moved by 10 mm (equal to the size of the stamp), the gap between the patterns is 30 µm, as shown in Figure 7a. Subsequently, when the stage is moved by 9.97 mm, the interval between the patterns is 10 µm. Figure 7a,b demonstrate that the gap between the patterns is reduced by approximately one third when the stage is moved by 9.97 mm compared to when it is moved by 10 mm. Finally, Figure 7c shows the results obtained when the stage moves 9.95 mm. When the stamp is moved by 9.95 mm, the patterns in the first process and the pattern in the next process overlap with each other, so each edge is broken. The pattern at the edge is broken, so when the overall process was carried out, the results show that the pattern was broken compared to the initial appearance of the stamp.

## 4. Discussion

The objectives of this experiment were to transfer the nanostructures onto large-area substrates using the step-and-repeat process and to reduce the stitch gap between pat-terns.

In the past, for large-area nanostructure transfer, the transfer was possible only when a stamp suitable for the size of the substrate was produced. This method has the disadvantages of increasing the difficulty of stamp production and increasing the cost. To compensate for these shortcomings, in this experiment, the size of the stamp was made small, and the nanostructures were transferred to a large-area substrate using the step-and-repeat process. After making a stamp using glass, nanopillars with a width of 600 nm, intervals of 400 nm, and a height of 270 nm were fabricated in an area of 10 mm × 10 mm. In this experiment, the process was carried out after the entire UV resin was coated. Although the entire area of the substrate was coated with UV resin, the transfer of the pattern occurred only on the surface where the substrate and the stamp were in contact. Using one stamp, it was possible to transfer the nanostructure to a 4-inch substrate through a total of 25 iterative processes, and as a result, it was confirmed that the pattern was uniformly transferred to the substrate. There was no significant difference between the results of the first and last processes after the processes were completed, as no damage to the stamp occurred during the experiment. Based on these results, it is expected that with the step-and-repeat NIL equipment used in this experiment, the pattern shape of the stamp will not be damaged and the nanostructure can be transferred uniformly to the area of the substrate, even if a small stamp is used.

When a stitch gap is employed, parts other than the pattern are pre-cured during the first process, so that a space is created when the second process is performed. As this stitch gap is minimized, the uniformity of the pattern can be improved during the process, and performance degradation can be prevented during application. The purpose of this experiment was to minimize this stitch gap. Experiments were conducted to minimize the stitch gap by setting the UV light in the equipment, making the stamp, and controlling the position of the stamp during the process. Firstly, the experimental setup was designed so that the UV light descends vertically and uniformly within the equipment. By preventing the UV light from coming down vertically and pre-curing the UV resin in the surrounding area, it was possible to continue with a process that could further reduce the stitch gap. In addition, during the process, UV light may leak through the step between the patterned and non-patterned areas, so it is important to minimize this part when producing a stamp. As the light leaking from the side in the step can temporarily harden the UV resin and affect the results of the next process, the step difference was 5 μm in this experiment, and the stamp was produced so that the minimum amount of UV light leaked during the process.

By controlling the movement position of the stamp during the process, the stitch gap could be minimized. When the stage movements were 10 and 9.95 mm, the stitch gaps were 30 and 10 μm, respectively. However, when the movement of the stage was set to 9.95 mm, the patterns overlapped between the first process and the next process, resulting in the pattern itself not being completely transferred. Before proceeding with the experiment, if the stamp is moved by as much as the pattern area of the stamp, there will theoretically be no overlap between the patterns and the stitch gap will be minimal. However, after conducting the experiment and looking at the results, when the stamp was moved by 9.97 mm, the stitch gap was reduced further compared to when the stamp was moved by 10 mm. Glass was used to produce the stamp, and if the pattern was formed by dicing the glass, the sharpness of the edge decreased due to the edge chamfer effect in the process. The sharpness of the edge was estimated to be approximately 10 μm, in reference to the previous procedure. According to the experimental results, the smallest stitch gap was obtained when the stamp position was moved by 9.97 mm. Furthermore, when the stamp was moved by 9.95 mm, the pattern overlapped completely, resulting in the structure shape not being transferred properly at the edge. When the stamp was moved by 10 mm, the stitch gap showed the largest gap. Consequently, the stitch gap was minimized when the stamp was moved by 9.97 mm rather than when moved by 10 mm, which is the size of the pattern area, due to mechanical factors that occur during stamp production.

## 5. Conclusions

Conventional NIL can efficiently and uniformly transfer nanostructures onto substrates, but the substrate size is limited, making it difficult to transfer nanostructures onto large-area substrates. In addition, as the substrate size increases, the stamp size must also increase, so there is a disadvantage that not only the cost but also the manufacturing difficulty increases when manufacturing the stamp.

In this study, instead of making a stamp suitable for the substrate size, an experiment was conducted to transfer nanostructures onto large areas using small stamps using the step-and-repeat NIL process. With a pattern area of 10 mm × 10 mm on the stamp, it was possible to transfer the nanostructures onto a 4-inch substrate through curing and demolding a total of 25 times. Consequently, even if the process was repeated with one stamp, there were no changes in the nanostructure shapes of the produced stamps or the nanostructures after the process. In addition, even after repeating the process several times and comparing the resulting structures, there were no significant differences in the pattern shapes. Another purpose of this study was to reduce the stitch gap using the direction of UV light, stamp production, and position control in the system. When curing occurs during the process, UV light comes down uniformly and vertically, and the light does not leak around the pattern area of the stamp, reducing the curing around it and minimizing the stitch gap. After moving the stamp by 10, 9.97, and 9.95 mm, it was found that it is possible to minimize the stitch interval by moving the position by 9.97 mm.

In the future, it will be possible to transfer nanostructures by increasing the substrate size, even if the stamp size is not made large, by referring to the results of this study. Furthermore, it is expected not only that the nanostructures will be transferred to the substrate but also that the nano–micro fusion structure will be possible, and it is expected that it will be possible to transfer the nanostructures to non-flat substrates or various substrate shapes.

## Data Availability

Not applicable.
